# Type II fatty acid synthesis is essential only for malaria parasite late liver stage development

**DOI:** 10.1111/j.1462-5822.2008.01270.x

**Published:** 2008-12-18

**Authors:** Ashley M Vaughan, Matthew T O'Neill, Alice S Tarun, Nelly Camargo, Thuan M Phuong, Ahmed S I Aly, Alan F Cowman, Stefan H I Kappe

**Affiliations:** 1 Seattle Biomedical Research InstituteSeattle, WA 98109, USA; 2 The Walter and Eliza Hall Institute of Medical ResearchMelbourne, Victoria, Australia; 3 Department of Global Health, University of WashingtonSeattle, WA 98195, USA

## Abstract

Intracellular malaria parasites require lipids for growth and replication. They possess a prokaryotic type II fatty acid synthesis (FAS II) pathway that localizes to the apicoplast plastid organelle and is assumed to be necessary for pathogenic blood stage replication. However, the importance of FAS II throughout the complex parasite life cycle remains unknown. We show in a rodent malaria model that FAS II enzymes localize to the sporozoite and liver stage apicoplast. Targeted deletion of *FabB/F*, a critical enzyme in fatty acid synthesis, did not affect parasite blood stage replication, mosquito stage development and initial infection in the liver. This was confirmed by knockout of *FabZ*, another critical FAS II enzyme. However, FAS II-deficient *Plasmodium yoelii* liver stages failed to form exo-erythrocytic merozoites, the invasive stage that first initiates blood stage infection. Furthermore, deletion of *FabI* in the human malaria parasite *Plasmodium falciparum* did not show a reduction in asexual blood stage replication *in vitro*. Malaria parasites therefore depend on the intrinsic FAS II pathway only at one specific life cycle transition point, from liver to blood.

## Introduction

Malaria parasites are protists belonging to the genus *Plasmodium*. They are obligate intracellular parasites that have two distinct replicating life cycle forms in the mammalian host. A massive one-time replication occurs in the liver after inoculation of sporozoite stages by the bite of an infected mosquito and results in the production and release of tens of thousands infectious exo-erythrocytic merozoites (Prudencio *et al*., 2006). These merozoites infect red blood cells and initiate the cyclic replication that occurs within the blood stream. Blood stage infection leads to malaria disease with *Plasmodium falciparum* alone afflicting more than 500 million people annually (Snow *et al*., 2005). *Plasmodium* replication in red blood cells produces between 8 and 36 merozoites with each invasive cycle (Cowman and Crabb, 2006) whereas one-time replication in the infected hepatocyte produces up to 40 000 merozoites (Shortt *et al*., 1951) – an ∼2000-fold difference. It is currently not well understood to what extent malaria parasites rely on parasitic scavenging of nutrients versus intrinsic synthesis for growth and replication.

**Fig. 1:**
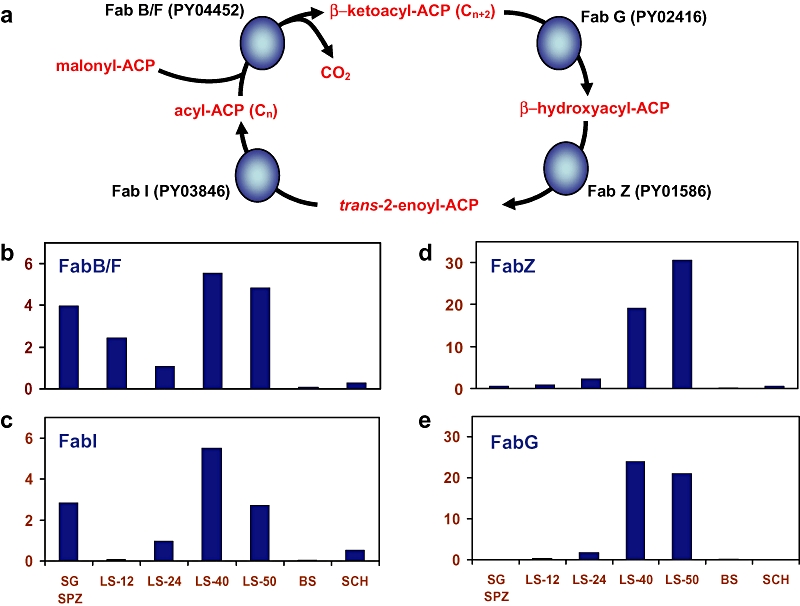
Transcript abundance of genes involved in the extension step of type II fatty acid synthesis (FAS II) in *Plasmodium yoelii*. (A) FAS II takes place by the condensation of malonyl-ACP with acyl-ACP to form β-ketoacyl-ACP and CO_2_. This reaction is catalysed by 3-oxoacyl-ACP synthase I/II (FabB/F, *P. yoelii* PlasmoDB identifier PY04452). β-Ketoacyl-ACP is reduced by β-ketoacyl-ACP reductase (FabG, PY02416) to form β-hydroxyacyl-ACP, dehydrated by β-hydroxyacyl-ACP dehydratase (FabZ, PY01586) to form *trans*-2-enoyl-ACP and finally reduced by enoyl-acyl carrier reductase (FabI, PY3846) to acyl-ACP. Successive cycles utilizing these four enzymes add two carbon units per cycle. To quantitatively determine the transcript levels of genes encoding the enzymes involved in FAS II, RNA was extracted from different stages of the *P. yoelii* life cycle, reverse transcribed and used for quantitative PCR. Expression levels were measured in salivary gland sporozoites (SG SPZ), mixed blood stages (BS), blood stage schizonts (SCH), and in liver stages 12, 24, 40 and 50 h after salivary gland sporozoite infection (LS-12, LS-24, LS-40 and LS-50 respectively). The expression profile for (B) FabB/F, (C) FabI, (D) FabZ and (E) FabG are shown. Note that for all four genes, expression is highly upregulated in liver stages.

Lipids are not only essential but are one of the most abundant components of all organisms and the malaria parasite needs a plentiful supply of lipids – specifically fatty acids for the membrane biogenesis necessary for invasive stage formation. *Plasmodium* parasites were initially assumed to lack the ability to synthesize their own fatty acids and thus rely on their hosts for lipid scavenging (Vial and Ancelin, 1992). However, this model came into question with the discovery of the apicoplast, a relict plastid organelle of *Plasmodium* (Kohler *et al*., 1997). Plant and algal plastids harbour several key biosynthetic pathways and the sequencing of the *P. falciparum* genome (Gardner *et al*., 2002) coupled with a detailed analysis of the proteins of known function that were targeted to the apicoplast (Foth *et al*., 2003) allowed the construction of an apicoplast-specific metabolic map (Ralph *et al*., 2004). The apicoplast is of cyanobacterial origin and one such apicoplast-targeted pathway is bacterial-like type II fatty acid synthesis (FAS II) (Waller *et al*., 1998), a *de novo* pathway by which *Plasmodium* can synthesize fatty acids from derivatives of acetate and malonate. The fatty acid chain extension step of FAS II is catalysed by four key enzymes – FabB/F, FabG, FabI and FabZ and the substrate/product of each reaction is covalently bound to the acyl carrier protein (ACP) cofactor (Fig. 1A). Conversely, the mammalian FAS I pathway utilizes a single enzyme complex and is not present in *Plasmodium* based on genome sequence analysis (Bahl *et al*., 2003). This is not the case for all apicomplexan parasites – the genome of *Toxoplasma gondii* encodes both FAS I and FAS II enzymes, *Cryptosporidium parvum* has FAS I enzyme whereas *Theileria annulata* does not harbour either FAS I or FAS II pathways (Mazumdar and Striepen, 2007). Deletion of ACP from *T. gondii* has demonstrated that apicoplast fatty acid synthesis is essential for organelle biogenesis and parasite survival in this parasite (Mazumdar *et al*., 2006).

The four *Plasmodium* FAS II enzymes are promising drug targets because they are of bacterial origin. The *P. falciparum* enzymes have been expressed *in vitro* and used to reconstitute the elongation module of FAS II (Sharma *et al*., 2007). The *in vitro* system mimicked the *in vivo* machinery and known inhibitors of the enzymes of the elongation module caused the expected accumulation of intermediates. Thus, *Plasmodium* possesses a functional FAS II pathway. An early study identified a *Plasmodium* FabI and showed that the FabI inhibitor triclosan kills blood stage parasites (Surolia and Surolia, 2001) and subsequently a significant effort has been undertaken to develop blood stage FAS II inhibitors to treat malaria (Gornicki, 2003; Sato and Wilson, 2005; Wiesner and Seeber, 2005). Although the data suggested that FAS II is necessary for intra-erythrocytic replication, the expression of FAS II enzymes has not been studied throughout the complex infection cycle of the parasite and their importance in parasite progression throughout the life cycle remains unknown.

We recently carried out a liver stage transcriptome and proteome analysis in the model rodent malaria parasite *Plasmodium yoelii* and observed that (i) the transcription of FAS II genes was increased in liver stages when compared with blood stages; (ii) FAS II enzymes were present in the liver stage proteome; and (iii) hexachlorophene, an inhibitor of FabG, was able to inhibit liver stage development *in vitro* (Tarun *et al*., 2008). These results suggested that FAS II might be important for parasite liver infection. Here, we show that expression of FAS II only occurs during the pre-erythrocytic phases of parasite infection and this allowed unprecedented imaging of the sporozoite and liver stage apicoplast. Strikingly, gene knockouts of *FabB/F* and *FabZ*, two of the enzymes involved in fatty acid synthesis demonstrated that FAS II is critical for normal liver stage development but not for blood stage or mosquito stage development. Knockout parasites did not form the first generation of invasive merozoites, which are the critical parasite transition stages to move from the liver into the blood stream and initiate red blood cell infection. In addition, gene knockout of FabI from *P. falciparum*, a further enzyme involved in FAS II, demonstrated that FAS II is not critical for *P. falciparum* blood stage replication.

## Results

### The transcript abundance of *P. yoelii* FAS II genes is highly upregulated in late liver stage development

We used quantitative RT-PCR (qPCR) to show upregulation of FAS II genes in liver stages because our previous microarray analysis had indicated preferential expression in liver stages of *P. yoelii* (Tarun *et al*., 2008). Transcript abundance was analysed in the different *P. yoelii* life cycle stages for the four FAS II genes encoding the enzymes involved in fatty acid extension (Fig. 1A). For all four genes, the level of transcription was highly increased in pre-erythrocytic stages but the most consistent and substantial expression was seen in late liver stages when compared with blood stages (Fig. 1B to E). The qPCR data suggest that FAS II is induced in liver stages and might thus play an important role for liver stage development.

### *P. yoelii* FAS II enzymes are expressed in sporozoites and liver stages and localize to the apicoplast

**Fig. 2:**
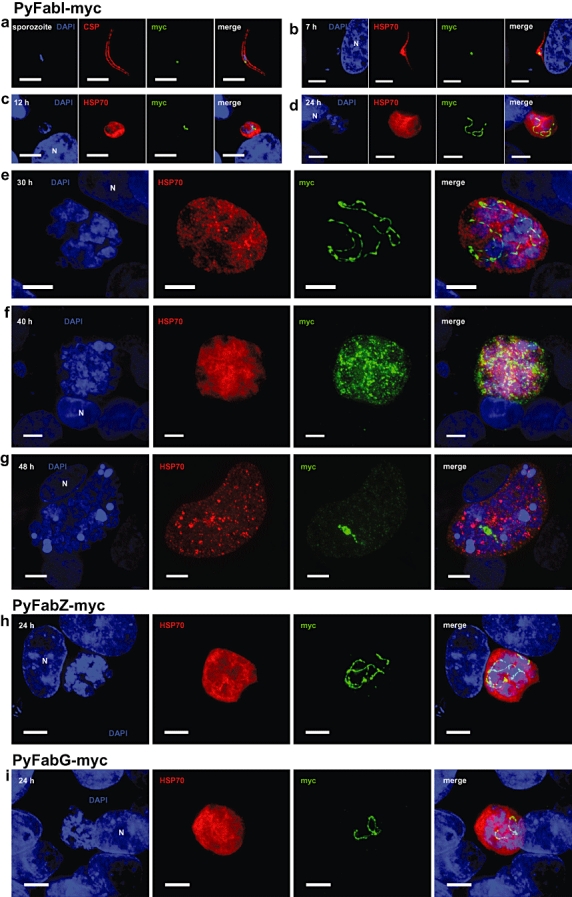
Expression of type II fatty acid synthesis enzymes involved in extension in salivary gland sporozoites and liver stages of *Plasmodium yoelii*. A transgenic *P. yoelii* parasite (PyFabI-myc) was generated expressing a second copy of *FabI* fused to a C-terminal quadruple-myc tag under the control of the *FabI* endogenous promoter. Expression of FabI in PyFabI-myc in life cycle stages of the parasite was monitored by IFA using a rabbit anti-myc antibody. Salivary gland sporozoites (A) were isolated from infected *Anopheles stephensi* mosquitoes, either fixed for IFA or used to infect HepG2:CD81 cells to study *in vitro* liver stage development. At increasing time points after infection (B, 7 h, C, 14 h, D, 24 h, E, 30 h, F, 40 h and G, 48 h) cells were fixed. Using the same methodology, PyFabZ-myc and PyFabG-myc transgenic parasites were created and expression of FabZ and FabG was monitored at 24 h after infection by IFA (H, PyFabZ and I, PyFabG). The salivary gland sporozoite was detected with a mouse anti-circumsporozoite protein (CSP) antibody and the liver stage cytoplasm was detected with a mouse anti-heat shock protein 70 (HSP70) antibody. Fluorescent staining was achieved with Alexa Fluor-conjugated secondary antibodies specific to rabbit (Alexa Fluor 488, green) and mouse (Alexa Fluor 594, red) IgG. Nuclear staining was achieved with DAPI. Fluorescent images were captured using deconvolution microscopy and a merge of captured images is presented on the far right pane (merge). The scale bars equal 5 μm and the white ‘N’ denotes the host cell nucleus. Note: As the liver stage progresses it dramatically increases in size. The results show that apicoplast-targeted FabI-myc is expressed in the salivary gland sporozoite and the developing liver stage (as are FabZ-myc and FabG-myc).

**Fig. 3:**
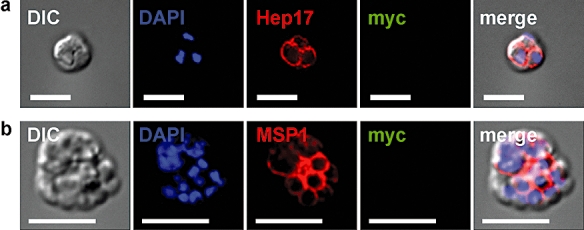
Lack of expression of quadruple-myc epitope-tagged FabI in the blood stages of the transgenic *P. yoelii* parasite PyFabI-myc. PyFabI-myc was generated to express a second copy of FabI under the control of its endogenous promoter with a C-terminal quadruple-myc tag. Expression of FabI in PyFabI-myc was monitored by IFA using an anti-myc antibody. Parasite blood stages were detected with antibodies to (A) the parasitophorous vacuole membrane protein Hep17 and (B) the merozoite-specific merozoite surface protein 1 (MSP1). Fluorescent staining was achieved with Alexa Fluor-conjugated secondary antibodies (Alexa Fluor 488, green and Alexa Fluor 594, red) specific to rabbit and mouse IgG. Nuclear staining was achieved with DAPI. Differential interference contrast (DIC) and fluorescent images were captured and processed using deconvolution microscopy and a merge of the captured images is presented on the far right pane (merge). Scale bar is 5 μm. FabI-myc expression was not detectable in blood stage of parasites.

To further investigate the expression of FAS II during the parasite life cycle we generated a transgenic *P. yoelii* line expressing a myc epitope-tagged FabI under the control of the endogenous *FabI* promoter (PyFabI-myc) (Fig. S1). The quadruple myc tag was fused to the carboxyl (C)-terminus of FabI and was followed by the 3′ UTR from *Plasmodium berghei* dihydrofolate reductase/thymidylate synthase (*DHFR/TS*). This expression cassette design should not alter the stage-specific expression of FabI as 3′UTRs are involved in mRNA stability, but not regulation of temporal expression. A similar strategy has previously been used to study the blood stage expression of *P. falciparum* subtilase 1 (Yeoh *et al*., 2007). PyFabI-myc allowed for the visualization of FabI expression throughout the parasite life cycle using indirect immunofluorescence assays (IFA) with anti-myc antibodies. FabI-myc expression was not detectable in the developing mosquito midgut oocysts and also not in oocyst sporozoites (Fig. S2). FabI-myc expression was first detected in salivary gland sporozoites and localized to a spherical structure close to the nucleus (Fig. 2A). All *P. yoelii* FAS II enzymes including *P. yoelii* FabI possess a bipartite leader sequence that predicts import into the apicoplast (data not shown). As FAS II enzymes localize to the apicoplast in the apicomplexan *T. gondii* (Waller *et al*., 1998), and FabI from the related apicocomplexan *Eimeria tenella* was localized to the apicoplast (Ferguson *et al*., 2007), we assume that the FabI-myc expression we detected in salivary gland sporozoites is apicoplast specific (Fig. 2A). This is the first published data showing the *Plasmodium* sporozoite apicoplast. To analyse FabI expression during liver stage development, HepG2:CD81 hepatoma cells (Silvie *et al*., 2003) were infected with sporozoites from the PyFabI-myc line. At 7 h post infection (pi) when intracellular sporozoites initiate transformation to trophozoites, apicoplast morphology as determined by FabI-myc staining (Fig. 2B), was similar to that of salivary gland sporozoites (Fig. 2A). At 14 h pi, parasite nuclear division commenced and the apicoplast initiated its division as indicated by the dumbbell shape (Fig. 2C). By 24 h pi, the apicoplast had formed a branched lariat-shaped structure (Fig. 2D), which became more elaborate with advanced liver stage development at 30 h pi (Fig. 2E). By 40 h pi (Fig. 2F) the apicoplast had differentiated into hundreds of intertwining structures that appeared to be segregating. These results show that FabI expression is initiated in salivary gland sporozoites and that there is robust apicoplast-specific FabI expression throughout liver stage development. Interestingly, the replication of the liver stage apicoplast bears striking similarities to that seen in the apicoplast of developing *P. falciparum* blood stages with reference to the changing appearance of the organelle during schizogony (van Dooren *et al*., 2005) but on a much more expansive scale. Strikingly, by 48 h pi (Fig. 2G), a time point when the *P. yoelii* liver stage schizont undergoes merozoite formation (Baer *et al*., 2007), FabI-myc expression was greatly reduced when compared with 40 h pi (Fig. 2F). This strongly suggests that FabI expression is downregulated shortly before or during exo-erythrocytic merozoite formation. Finally, we investigated whether FabI is expressed during asexual blood stage replication and found that FabI-myc expression was not detected in blood stages (Fig. 3). To further analyse FAS II expression, additional parasite lines were created that expressed epitope-tagged FabZ and FabG (PyFabZ-myc or PyFabG-myc). Similar expression patterns were seen throughout the life cycle when compared with FabI-myc (data not shown), exemplified by the labelling of a branched lariat-shaped structure at 24 h pi for both PyFabZ-myc and PyFabG-myc (Fig. 2H and I). Taken as a whole, the expression data led us to hypothesize that FAS II is not essential for blood stage development but might be critical for liver stage development.

### *P. yoelii* FabB/F and FabZ are not essential for blood stage growth

**Fig. 4:**
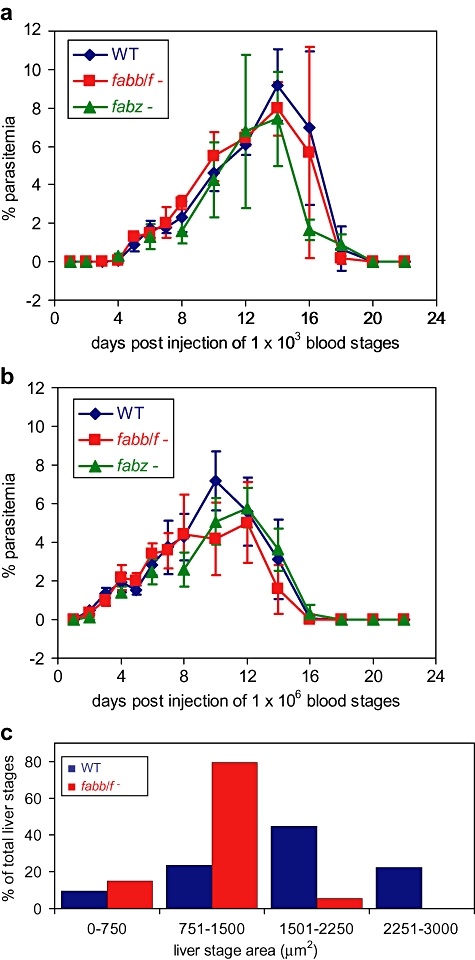
Normal blood stage growth of *P. yoelii fabb/f*^−^ and *fabz*^−^ parasites and impaired liver stage growth of *P. yoelii fabb/f*^−^ parasites. Double homologous cross-over recombination was used to delete *P. yoelii FabB/F* and *FabZ* to generate *P. yoelii fabb/f*^−^ and *fabz*^−^ blood stage parasites respectively. To assess blood stage growth, one thousand (A) or one million (B) WT, *fabb/f*^−^ clone 1 or *fabz*^−^ clone 1 blood stage parasites were injected iv into 6-week-old female SW mice (*n* = 4 for each group). Subsequently, blood stage parasitaemia was measured until parasite clearance by Giemsa-stained blood smear and expressed as a percentage. To assay liver stage growth, one million WT or *fabb/f*^−^ sporozoites isolated from the salivary glands of infected *Anopheles stephensi* mosquitoes were injected into BALB/c mice. The livers were removed 44 h pi, fixed and then cut into 50 μm sections. Liver stage parasites were detected using IFA utilizing an antibody to the parasitophorous vacuole membrane protein Hep17. (C) The area of the parasite at its widest diameter, based on scanning through the z plane of the section, was determined and expressed as a percentage of the total number of parasites in quartiles (for WT, *n* = 251 and for *fabb/f*^−^, *n* = 147). The results show that both FabB/F and FabZ are not essential for normal blood stage replication but FabB/F is essential for normal late liver stage development.

To test the hypothesis that FAS II is not essential for blood stage development, we deleted *P. yoelii FabB/F*, the enzyme that catalyses the condensation of malonyl-ACP with the lengthening fatty acyl-ACP formed by FabI (Fig. 1A) from the parasite genome using a double cross-over recombination strategy (Menard and Janse, 1997; Tarun *et al*., 2007). We decided to delete *FabB/F* because it catalyses an essential step in fatty acid synthesis. PCR genotyping using specific primers pairs confirmed the recombination event and the deletion of the *FabB/F* gene (Fig. S3) in two cloned knockout parasite lines (*fabb/f*^−^) from two independent transfections. To assay the effect of *FabB/F* deletion on blood stage development, mice were intravenously (iv) injected with 1 × 10^3^ or 1 × 10^6^*fabb/f*^−^ clone 1 parasites and blood stage development was followed over time in comparison with wild-type (WT) parasites (Fig. 4A and B). There was no significant difference in the growth rate of *fabb/f*^−^ clone 1 parasites and WT parasites. Normal clearance of the parasite that is observed for this non-lethal *P. yoelii* strain occurred for both WT and knockout approximately 20 days pi (Fig. 4A and B). The results demonstrate that the loss of *FabB/F* and therefore the loss of FAS II had no deleterious effect on *P. yoelii* asexual blood stage replication *in vivo*. Furthermore, the blood stage *fabb/f*^−^ parasites showed normal gametocyte development and male gamete exflagellation (data not shown). To confirm our observations with *fabb/f*^−^ parasites we used similar methodologies to generate a parasite with a deletion in a second synthesis enzyme, FabZ. FabZ catalyses the dehydration of β-hydroxyacyl-ACP to form *trans*-2-enoyl-ACP (Fig. 1A). PCR genotyping confirmed the creation of *fabz*^−^ parasites in two cloned knockout lines from independent transfections (Fig. S3). As for *fabb/f*^−^ parasites, the deletion of *FabZ* had no deleterious effect on the growth of *fabz*^−^ blood stage parasites (Fig. 4A and B). A similar blood stage phenotype from the deletion of a second enzyme in FAS II adds strength to our hypothesis that FAS II is not necessary for blood stage replication.

### *P. yoelii* FabB/F and FabZ are necessary for pre-erythrocytic stage infection

After transmission of either *fabb/f*^−^ or *fabz*^−^ to *Anopheles stephensi* mosquitoes, we observed normal development of mosquito midgut oocysts, formation of oocyst sporozoites and invasion of sporozoites into the salivary glands as indicated by the enumeration of salivary gland sporozoites in comparison with WT (Table S1: data for one clone for each deletion are shown). Therefore, FAS II is not necessary for parasite development in the mosquito. Next, salivary gland sporozoites were injected iv into BALB/c mice. Mice were injected with 10 000 and 50 000 (*n* = 8) knockout sporozoites or with 10 000 WT sporozoites. Mice were assayed for blood stage parasitaemia every other day from day 3 post injection (pi) until day 15 by Giemsa-stained blood smears (Table 1). After 3 days, all mice injected with WT sporozoites exhibited patent blood stage parasitaemia. Strikingly, none of the mice injected with 10 000 and 50 000 *fabb/f*^−^ sporozoites or *fabz*^−^ sporozoites became blood stage patent and this was true for both clones of each of the knockouts. These results demonstrate that the lack of FAS II renders the pre-erythrocytic parasite unable to successfully infect the mammalian host.

**Table 1:** *P. yoelii* FAS II is critical for successful pre-erythrocytic stage infection.

Parasite genotype	# sporozoites injected	# mice injected	# mice blood stage patent (day of patency)
Wild type	10 000	10	10(3)
*fabb/f*^−^ clone 1	10 000	18	0(–)^a^
*fabb*/*f*^−^ clone 1	50 000	18	0(–)^a^
*fabb*/*f*^−^ clone 2	10 000	12	0(–)^a^
*fabb/f*^−^ clone 2	50 000	12	0(–)^a^
*fabz*^−^ clone 1	10 000	8	0(–)^b^
*fabz*^−^ clone 1	50 000	8	0(–)^b^
*fabz*^−^ clone 2	10 000	4	0(–)^b^
*fabz*^−^ clone 2	50 000	4	0(–)^b^

a Mice injected with 10 000 and 50 000 *fabb*/*f*^−^ sporozoites were followed for 15 days post injection and never became blood stage patent.

b Mice injected with 10 000 and 50 000 *fabz*^−^ sporozoites were followed for 15 days post injection and never became blood stage patent.

Salivary gland sporozoites were injected intravenously into BALB/c mice and the mice were assayed for blood stage patency from the third day after injection.

### *P. yoelii fabb/f*^−^ parasites arrest late in liver stage development

**Fig. 5:**
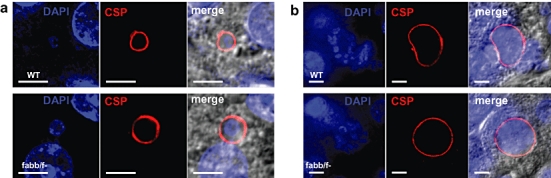
Development of *Plasmodium yoelii* WT and *fabb/f*^−^ early liver stages *in vivo.* Salivary gland sporozoites were isolated from *Anopheles stephensi* mosquitoes and injected iv into BALB/c mice (one million for both WT and *fabb/f*^−^). The livers were removed at (A) 12 h pi and (B) 24 h pi, fixed and cut into 50 μm sections. Liver stage parasites were detected using an IFA utilizing antibodies to the circumsporozoite protein (CSP). Fluorescent staining was achieved with Alexa Fluor-conjugated secondary antibodies specific to rabbit (Alexa Fluor 488, green) and mouse (Alexa Fluor 594, red) IgG. Nuclear staining was achieved with DAPI. Differential interference contrast and fluorescent images were captured and processed using deconvolution microscopy and a merge of the captured images is presented on the far right pane (merge). The scale bars equal 5 μm. Note: As the liver stage progresses there is no difference in the size between the WT and *fabb/f*^−^ parasite. The results suggest that FabB/F is not essential for early liver stage development.

**Fig. 6:**
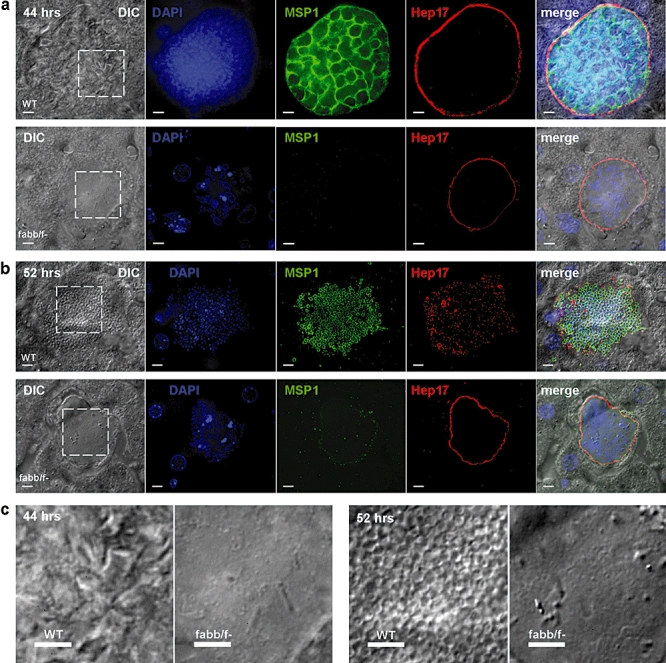
Development of *Plasmodium yoelii* WT and *fabb/f*^−^ late liver stages *in vivo.* Sporozoites were isolated and injected into mice as for Fig. 5. The livers were removed at (A) 44 h pi and (B) 52 h pi, fixed and cut into 50 μm sections. Liver stage parasites were detected using an IFA utilizing antibodies to the parasitophorous vacuole membrane protein Hep17 and MSP1. Fluorescent staining was achieved with Alexa Fluor-conjugated secondary antibodies specific to rabbit (Alexa Fluor 488, green) and mouse (Alexa Fluor 594, red) IgG. Nuclear staining was achieved with DAPI. Differential interference contrast (DIC) and fluorescent images were captured and processed using deconvolution microscopy and a merge of the captured images is presented on the far right pane (merge). The scale bars equal 5 μm. (C) Liver stage DIC images from (A) and (B) were magnified (dashed box) to show the difference in appearance between WT and *fabb/f*^−^ parasites. Note: The results show that deletion of *FabB/F* impairs late liver stage development as visualized by the decreased size of the liver stage at 44 and 52 h pi, the decreased nuclear division, the lack of MSP1 staining and the lack of merozoite differentiation. This suggests that type II fatty acid synthesis is essential for late liver stage development and the formation of infectious exo-erythrocytic merozoites.

To further investigate the phenotype of the knockout parasites we chose to follow the progression of *fabb/f*^−^ clone 1 parasites in the liver. BALB/c mice were iv injected with 1 × 10^6^ sporozoites (WT or *fabb/f*^−^) and sacrificed at different time points of liver stage development. The infected livers were perfused, removed, sectioned and parasite load and development was assayed by immunofluorescence microscopy. At 12 and 24 h pi *fabb/f*^−^ liver stages showed normal development that was indistinguishable from WT, i.e. they invaded hepatocytes, formed a parasitophorous vacuole (PV), transformed into trophozoites and initiated schizogony (Fig. 5A and B). However, by 44 h pi the size of the *fabb/f*^−^ liver stages was significantly less than that of WT (Figs 4C and 6A). Quantification of cell size showed that more than 60% of WT liver stages at this time point had a maximum area at their widest diameter of > 1500 μm^2^, whereas more than 90% of the *fabb/f*^−^ liver stages were less than 1500 μm^2^ (Fig. 4C). In addition, abnormal progression of nuclear division became apparent. Furthermore, unlike WT, *fabb/f*^−^ liver stages did not show merozoite surface protein 1 (MSP1) (Suhrbier *et al*., 1989) expression (Fig. 6A and B). At 52 h pi, most of the WT parasites had formed and released merozoites or were in the final stages of merozoite formation (Fig. 6B) but, although the *fabb/f*^−^ liver stages had somewhat increased in size in comparison with 44 h, there was still no significant expression of MSP1 (Fig. 6B). High-magnification differential interference contrast images of the liver stage parasites (Fig. 6C) showed that at 44 h pi, cytomere formation, which is due to multiple invaginations of the liver stage plasma membrane, was occurring in WT but not in the *fabb/f*^−^ parasites and at 52 h pi, merozoites had formed and segregated in WT liver stages but not in the *fabb/f*^−^ liver stages (Fig. 6C). The sporozoite infectivity and liver stage developmental data therefore suggest that *fabb/f*^−^ parasites are normal with regard to hepatocyte invasion and liver stage development up to a point in late liver stage schizogony. However, *fabb/f*^−^ liver stages do not reach maturity at the time point at which WT liver stages do and are unable to form infectious merozoites, explaining the lack of onset of blood stage infection from the liver. This is an unprecedented phenotype in late liver stage differentiation caused by the lack of an intrinsic metabolic pathway.

### *P. falciparum* FabI is not essential for blood stage growth

**Fig. 7:**
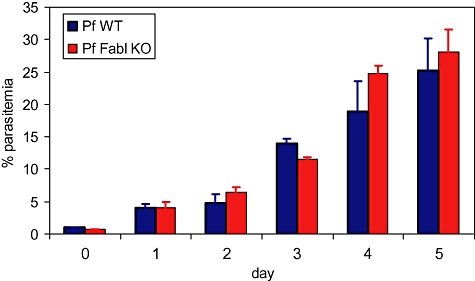
Normal blood stage growth of *P. falciparum fabi*^−^ parasites. Double homologous cross-over recombination was used to delete *P. falciparum FabI* and generate *P. falciparum fabi*^−^ blood stage parasites. To assess blood stage growth, parasite cultures of both the WT NF54 and the *fabi*^−^ clone E6 containing mainly ring stages were synchronized twice within 4 h using sorbitol (Lambros and Vanderberg, 1979). Parasite density was determined and the culture was diluted to 0.8% parasitaemia, 5% haematocrit. Cultures were maintained under an atmosphere of reduced oxygen at 37°C and medium was refreshed every 24 h. Growth, based on percentage parasitaemia, was monitored by Giemsa-stained thin blood smears every 24 h for 5 days. The experiment was carried out in triplicate and mean and standard deviations are shown. The results show that there are no significant differences in the growth of the two parasite lines.

To test whether the FAS II pathway might be dispensable for a clinically relevant human malaria parasite, we decided to study FabI in the NF54 strain of *P. falciparum*. We were able to delete the *P. falciparum FabI* gene from blood stage parasites using double homologous cross-over recombination in association with positive and negative selection. Deletion of the gene was confirmed by Southern blot analysis of the parental line (data not shown) and two clonal lines derived from the parental line (Fig. S4). We then compared the replication of the *P. falciparum fabi*^−^ blood stages with that of the WT NF54 strain. We saw no significant differences in replication efficiency between WT and *fabi*^−^ (Fig. 7 and data not shown) in the growth assay, which strongly suggests that, as for *P. yoelii*, the FAS II pathway is dispensable for *P. falciparum* blood stage growth.

## Discussion

Although many metabolic functions have been assigned to the *Plasmodium* apicoplast (Ralph *et al*., 2004), including FAS II, it is not known if these pathways are essential for every life cycle stage of the malaria parasite. As the parasite inhabits both extracellular and intracellular niches and replicates in the mosquito vector and mammalian host, its ability to scavenge host nutrients to support replication presumably varies greatly depending on its environment. The study presented herein analyses for the first time the importance of a metabolic pathway throughout the parasite life cycle. We demonstrated that apicoplast-targeted FAS II is only necessary for *Plasmodium* late liver stage development. Thus, in all other life cycle stages either the parasite synthesizes fatty acids by a yet unidentified *de novo* pathway or the parasite is able to scavenge all the fatty acids it requires from the host. Moreover, FAS II is only needed late in liver stage schizogony. We have previously shown that the liver stage PV membrane-resident protein UIS3 interacts with the hepatocyte lipid carrier liver-fatty acid binding protein (Mikolajczak *et al*., 2007). It is possible that this interaction allows for the transfer of lipids from the hepatocyte cytosol to UIS3 and subsequently to the developing liver stage and this concept has recently been buoyed by the fact that *P. falciparum* UIS3 cocrystallizes with the lipid phosphatidylethanolamine (Sharma *et al*., 2008). Thus, the parasite liver stage might have developed a means of directly transferring lipids from its host. Nevertheless, host lipids alone are clearly not sufficient for completion of liver stage development. In the malaria model under study, no difference was seen in the first half of liver stage development (up to 24 h pi) between WT and *fabb/f*^−^ parasites and it was only after 24 h that the *fabb/f*^−^ liver stages showed growth retardation, accompanied by lack of cytomere formation and subsequent merozoite differentiation. Thus, during the complete *P. yoelii* life cycle, FAS II is required only for the final stages of parasite transition from its first site of infection in the liver to the blood. We do not currently know why FAS II is only necessary for late liver stage development. It is possible that the sheer amount of membrane biogenesis required for the formation of tens of thousands of merozoites (Baer *et al*., 2007) cannot be met by host lipid scavenging and thus also relies on parasite-derived fatty acid synthesis to give a final boost to the formation of the merozoite membrane phospholipid bilayers. Alternatively, FAS II could be necessary to provide a particular fatty acid that is necessary for late liver stage development. In the sleeping sickness parasite, *Trypanosoma brucei*, the bloodstream form evades the host's immune response by expressing continually switching variant surface glycoprotein molecules (Donelson, 2003). The variant surface glycoprotein is attached to the plasma membrane by a glycosylphosphatidylinositol (GPI) anchor whose fatty acids are exclusively myristate (Ferguson and Cross, 1984). The continuous supply of myristate cannot be met by the bloodstream and *T. brucei* has a unique *de novo* fatty acid synthesis pathway to supply its myristate needs (Lee *et al*., 2006). Perhaps *Plasmodium* FAS II is fulfilling a similar need by supplying particular fatty acids necessary for late liver stage schizogony that the parasite cannot obtain from its host. It is of interest to note that MSP1 is a GPI-anchored protein (Gerold *et al*., 1996). However, we currently do not know if GPI biosynthesis is abrogated in FAS II-deficient liver stages. Notwithstanding, we observed lack of MSP1 in liver stages of FAS II knockout parasites, suggesting that MSP1 could play an essential role in the formation of exo-erythrocytic merozoites.

Our data show that some FAS II enzymes are initially expressed in the salivary gland sporozoite based on both epitope tagging of FabI and our qRT-PCR data. This also revealed a high transcript abundance for *FabI* and *FabB/F* in salivary gland sporozoites although this was not the case for *FabG* and *FabZ*. We currently do not understand the functional significance of these increases; nevertheless, the pathway is clearly not necessary in this life cycle stage as both *fabb/f*^−^ and *fabz*^−^ sporozoites infected the mosquito salivary glands and were able to initiate liver stage infection. Previously, it was shown that depletion of UIS3 and UIS4 (Mueller *et al*., 2005a,b), proteins that are initially expressed in sporozoites and localize to the PV membrane during liver stage development, as well as the sporozoite proteins P52 and P36 (van Dijk *et al*., 2005; Ishino *et al*., 2005; Labaied *et al*., 2007), and the sporozoite asparagine-rich protein 1 (Aly *et al*., 2008), cause arrest early in liver stage development. However, depletion of these proteins, as shown here for FabB/F and FabZ, does also not affect salivary gland sporozoite maturation. Furthermore, the liver stage phenotype for *fabb/f*^−^ parasites is unique, when compared with the above as the parasites do not arrest early in liver stage development but grow substantially and undergo schizogony. At 52 h pi, the *fabb/f*^−^ liver stages still seemed viable, based on the presence of an intact PV membrane (visualized by Hep17 expression). Nevertheless, liver stage development is not completed and the parasites might be cleared by host defence mechanisms although this requires further investigation.

Our studies have also shown that FAS II is not necessary for either the mosquito stage or blood stage of the *P. yoelii* life cycle. It is currently not known how the parasite utilizes host lipids whilst developing on the mosquito midgut but it is well documented that host serum fatty acids are utilized by parasite blood stages for growth (Krishnegowda and Gowda, 2003; Mi-Ichi *et al*., 2006). Our results appear to contradict previous work demonstrating that triclosan, an inhibitor of FabI (McMurry *et al*., 1998), is able to kill cultured *P. falciparum* blood stages and *in vivo* rodent blood stage infections of *P. berghei* (Surolia and Surolia, 2001). Others have also concluded that FAS II inhibitors are directly interacting with their apicoplast targets in *Plasmodium* blood stages (Surolia *et al*., 2004; Jones *et al*., 2005; Tasdemir *et al*., 2006; Goodman *et al*., 2007), thereby inhibiting parasite growth. However, it is possible that the FAS II inhibitors used are having off target effects on parasite growth. This has previously been shown in *T. brucei*, where it was concluded that triclosan killing may be due to a non-specific perturbation of subcellular membrane structure leading to dysfunction in sensitive membrane-resident biochemical pathways (Paul *et al*., 2004). Furthermore, studies of the effect of triclosan on several microorganisms have concluded that the interaction of triclosan with the bacterial cell is complex and its lethality cannot be explained solely by the inhibition of metabolic pathways such as FAS II (Escalada *et al*., 2005). Thus, triclosan could be killing *P. falciparum* blood stages by inhibiting a vital process other than FAS II. It has recently been shown based on transcriptional data obtained form malaria patient isolates, that there appears to be three distinct *P. falciparum* blood stage physiological states (Daily *et al*., 2007). These three states closely resemble (i) active growth; (ii) starvation; and (iii) environmental stress. In the starvation state, the authors noted an upregulation of FAS II genes when compared with active growth. These data suggest that under active glycolytic growth conditions, which were similar to the *P. falciparum* 3D7 growth conditions *in vitro* for which transcriptome profiles have been previously published (Bozdech *et al*., 2003; Le Roch *et al*., 2003), FAS II is not significantly involved in blood stage replication.

This study has concentrated on the effect of FAS II depletion on liver stage development in the rodent malaria parasite *P. yoelii.* However, we have also shown that the deletion of *FabI* from the human malaria parasite, *P. falciparum* has no apparent effect on blood stage replication when compared with WT parasites. This result, which converges on the results we obtained for the deletions of *P. yoelii FabB/F* and *FabZ*, demonstrates that FAS II is not required in blood stages. Future work might determine whether deletion of *FabI* affects *P. falciparum* sporozoite infectivity in humans; however, this requires a clinical investigation. Collectively, the data suggest that the metabolic pathways present in the *Plasmodium* apicoplast are not always necessary to support parasite progression through the specific parts of the life cycle. Thus, this unique organelle is likely to perform its critical metabolic functions at only certain time points during the *Plasmodium* life cycle and the functions it performs will be directly related to the needs the parasite cannot fulfil by nutrient uptake from the host. The *Plasmodium* apicoplast, as well as being the centre for FAS II, is also thought to harbour the only pyruvate dehydrogenase complex the parasite possesses (Foth *et al*., 2005). It is possible that pyruvate dehydrogenase is solely required by the apicoplast for the formation of acetyl CoA which is subsequently utilized by FAS II. Further studies are needed to address this issue.

Although our findings concerning liver stage development were generated with the rodent malaria parasite *P. yoelii*, the high conservation of FAS II among *Plasmodium* species (Carlton *et al*., 2002) suggests that FAS II is also essential for *P. falciparum* liver stage development. This might have consequences for the direction of antimalaria FAS II inhibitor drug development. Rather than concentrating on the *P. falciparum* blood stage, research into FAS II inhibitors should concentrate on their efficacy against the initial, clinically silent liver stage of infection. This might significantly contribute to the goal of eradicating malaria.

## Experimental procedures

### Experimental animals

Six- to eight-week-old female Swiss Webster (SW) mice and female BALB/c mice were purchased from Harlan (Indianapolis, IN). Animal handling was conducted according to institutional animal care and use committee-approved protocols.

### Parasite isolation

*Plasmodium yoelii* (17XNL) liver stage-infected hepatocytes were isolated at four time points post infection from infected mice: 12 h (LS-12), 24 h (LS-24), 40 h (LS-40) and 50 h (LS-50). Sporozoites were isolated from mosquito salivary glands at day 15 after infectious blood meal. Contaminating mosquito tissue was removed from sporozoite preparation by passing the extract over a DEAE cellulose column. For the preparation of parasites in the mixed blood stages, blood was harvested from infected SW mice when parasitaemia was at 5–10%. Lymphocytes were removed by passing the infected blood through a sephadex column. Purified blood stage schizonts were prepared from Nycodenz purification of *P. yoelii* infected blood cultured for 12 h.

### Quantitative real-time PCR

Total RNA from each sample was extracted using Trizol (Invitrogen) and DNase treated using Turbo-DNA free (Ambion). Total RNA was then subjected to two rounds of linear amplification using the Amino Allyl Message Amp II aRNA Amplification Kit (Ambion) according to manufacturer's directions. First-strand cDNA was synthesized from 500 ng of amplified RNA (aRNA) using the Superscript III Platinum RT kit (Invitrogen). The resulting cDNA was diluted 1:5 with nuclease-free water. Primers (Table S2) were designed using Primer Express v3.0 (Applied Biosystems). Designs were based on the mRNA sequence of the genes available at PlasmoDB. Amplicons were set to be between 100 and 200 bp. Real-time PCR analysis was performed on ABI prism 7300 Sequence Detection Systems using the SYBR Green PCR Master Mix (Applied Biosystems). The PCR reaction consisted of 12.5 μl of SYBR Green PCR Master Mix, 20 pmole of forward and reverse primers and 5 μl of diluted cDNA in a total volume of 25 μl. PCR cycling conditions were performed using the default conditions of the ABI Prism 7300 SDS Software.

Quantification of gene expression was done using the Relative Standard Curve Method (Applied Biosystems bulletin). The standard is prepared from a mixture of aRNA (salivary gland sporozoites, blood stages and liver stages) in a 1:1:1 ratio. First-strand cDNA is prepared from the standard and dilutions of 1:1, 1:5, 1:10, 1:20 and 1:50 of the resulting cDNA was used as templates for real-time PCR for each primer pair. The relative quantity of gene in the cDNAs from the seven aRNA samples (salivary gland sporozoites, LS-12, LS-24, LS-40, LS-50, mixed blood stages and blood stage schizonts) is interpolated from the corresponding standard curve. Expression of the FAS II genes was normalized to the expression of three housekeeping genes: *P. yoelii* 18S, 14-3-3 protein (PY01841) and the mitochondrial EF-TU gene (PY06134) in each cDNA sample. Normalized quantity of each target gene is expressed as the ratio of the relative amount of target gene over the average quantity of the three housekeeping genes.

### Generation of *P. yoelii* transgenic parasites expressing FabI-myc, FabZ-myc and FabG-myc

To epitope tag FabI, a quadruple (4×) myc tag sequence followed by a stop codon was introduced into the b3D.DT^H.^D vector (Catalog # MRA-80 in the MR4 Malaria Research and Reference Reagent Resource Center; http://www.mr4.org). A graphic representation of the construction of the plasmid and its subsequent transfection into *P. yoelii* blood stages is given in Fig. S1. Approximately one kilobase pairs (kb) of the 3′ untranslated region of the *P. berghei DHFR/TS* gene was added to the C-terminus of the 4× myc tag to ensure stability of the recombinant messenger RNA. The *P. yoelii FabI* gene (PY03846), including approximately 1 kb of sequence upstream of the start codon was amplified from *P. yoelii* 17XNL genomic DNA and cloned in frame (without the stop codon) and upstream of the 4× myc tag. The resulting plasmid was linearized with BsaI for integration into *P. yoelii* 17XNL blood stage schizonts using standard procedures (Labaied *et al*., 2007). Integration of the plasmid to create PyFabI-myc gave rise to a parasite line that expressed two copies of FabI, both with the endogenous promoter and one containing the 4× myc epitope tag. Oligonucleotide primers used are in Table S2. A similar strategy was used to generate PyFabZ-myc and PyFabG-myc.

### *In vitro* analysis of PyFabI-myc, PyFabG-myc and PyFabZ-myc liver stages

*In vitro* assays were conducted using the human hepatoma cell line HepG2 expressing the tetraspanin CD81 (HepG2:CD81) cultured in Dulbecco's modified Eagle's medium with 10% fetal calf serum at 37°C and 5% CO_2_. Infections were done by adding 5 × 10^4^ sporozoites to individual chambers of an 8 well chamber slide (Laboratory-Tek Permanox eight-well chamber slide; Nalge Nunc International, Rochester, NY) which had been seeded with 10^5^ subconfluent HepG2:CD81 cells the previous day. The slide was then centrifuged at 500 *g* for 2.5 min to aid sporozoite infection. Sporozoites which had failed to invade cells were removed after 2 h and the media were replaced. For the liver stage development assay, infections were maintained for various time periods after the addition of salivary gland sporozoites. Subsequently the infected cells were fixed in 4% paraformaldehyde in phosphate buffered saline (PBS) for 10 min, then blocked and permeabilized in PBS with 2% bovine serum albumin and 0.2% Triton-X 100 (PBS/BSA/Triton) for IFA. IFA was carried out in PBS/BSA/Triton. The double staining was performed using a mouse monoclonal anti-*P. berghei* HSP70 primary antibody (Tsuji *et al*., 1994), a parasite cytoplasmic marker and a rabbit polyclonal anti-myc antibody (Santa Cruz Biotechnology, Santa Cruz, CA), which recognizes the recombinant apicoplast-expressed FabI-myc. Fluorescent staining was achieved with Alexa Fluor-conjugated secondary antibodies (Invitrogen Corporation, Carlsbad, CA) specific to rabbit (Alexa Fluor 488, green) and mouse (Alexa Fluor 594, red) IgG. Cells were stained with 4′,6′-diamidino-2-phenylindole (DAPI) to visualize the DNA and mounted with FluoroGuard anti-fade reagent (Bio-Rad, Hercules, CA). Preparations were analysed using a fluorescence inverted microscope (Eclipse TE2000-E; Nikon), and images were acquired using Olympus 1 × 70 Delta Vision deconvolution microscopy.

### Generation of P*. yoelii fabb/f*^−^ and *fabz*^−^ parasites

For the targeted deletion of the *FabB/F* (PY04452) and *FabZ* (PY01586) genomic loci, two DNA fragments containing approximately 0.8 kb of the 5′UTR and 3′UTR of the gene were amplified using *P. yoelii* 17XNL genomic DNA as a template. The two fragments were cloned into the b3D.DT^H.^D targeting vector between the *T. gondii DHFR/TS* gene, which allows selection of recombination events with pyrimethamine. The plasmid was transfected into *P. yoelii* 17XNL blood stage schizonts using standard procedures (Labaied *et al*., 2007). Two independent clones of *fabb/f*^−^ and *fabz*^−^ parasites were obtained by limited dilution of parentals from independent transfection experiments. A graphic representation of the construction of the plasmid and its subsequent transfection into *P. yoelii* blood stages is given in Fig. 3 and the primers used in Table S2.

### Phenotypic analysis of blood stage *P. yoelii fabb/f*^−^ and *fabz*^−^ parasites

To assay growth of non-lethal *P. yoelii* 17XNL WT, *fabb/f*^−^ and *fabz*^−^ blood stages, blood was removed from infected SW mice when parasitaemia was between 0.5% and 1.5%. The blood was diluted in RPMI-1640 media (HyClone, Logan, UT) so that 100 μl contained either 10^3^ or 10^6^ parasites. SW mice (four in each group) were then injected iv with 10^3^ or 10^6^ parasites (WT or *fabb/f*^−^). Percentage parasitaemia was followed as often as daily until clearance, by assay of Giemsa-stained blood smear.

### Phenotypic analysis of *P. yoelii fabb/f*^−^ and *fabz*^−^ parasites in the mosquito

*Anopheles stephensi* mosquitoes were infected with *P. yoelii* WT*, fabb/f*^−^ or *fabz*^−^ parasites by blood feeding for 6 min on the first and second day using infected SW mice and subsequently maintained under a cycle of 12.5 h light/11.5 h dark and 70% humidity at 24.5°C. Gametocyte exflagellation capacity was evaluated microscopically before mosquito blood meal. Infected mosquitoes were dissected (at least 20 mosquitoes for each dissection) at days 10 and 14 (after the first infectious blood meal) to determine the presence of midgut oocyst sporozoites and the numbers of salivary gland sporozoites respectively.

### *In vivo* analysis of *P. yoelii fabb/f*^−^ liver stage development

To analyse *in vivo* sporozoite infection and liver stage development, BALB/c mice were injected iv with 10^6^ WT or *fabb/f*^−^ sporozoites. For each parasite population, the livers were harvested from euthanized mice at several time points post infection (12, 24, 44 and 52 h). Livers were perfused with PBS, washed extensively with PBS and then fixed in 4% paraformaldehyde. Liver lobes were cut into 50 μm sections using a Vibratome apparatus (Ted Pella Inc., Redding, CA). For IFA, sections were permeabilized in Tris buffered saline (TBS) containing 3% H_2_O_2_ and 0.25% Triton X-100 for 30 min at room temperature. Sections were then blocked in TBS containing 5% dried milk (TBS-M) at least 1 h and incubated with primary antibody in TBS-M at 4°C overnight. Primary antibodies used were mouse monoclonal anti-circumsporozoite protein, mouse monoclonal anti-Hep17 (Charoenvit *et al*., 1995) and rabbit polyclonal anti-MSP1. After washing in TBS, secondary antibody was added in TBS-M for 2 h at room temperature in a similar manner as above. After further washing, the section was incubated in 0.06% KMnO_4_ for 10 min to quench background fluorescence. The section was then washed with TBS and cells were stained with DAPI to visualize the DNA and mounted with FluoroGuard anti-fade reagent (Bio-Rad, Hercules, CA). Preparations were analysed as above for fluorescence with the addition of acquisition of a differential interference contrast image.

### Comparison of WT and *fabb/f*^−^ liver stage growth

To compare the sizes of parasite liver stages at 44 h pi, liver sections labelled with Hep17 (see above) were sequentially scanned using Nikon fluorescence microscopy. The greatest diameter for each liver stage detected was determined by adjusting the z plane of the liver section and the area of the parasite was subsequently determined. For the WT, 251 liver stages were assayed and for the *fabb/f*^−^, 147. All parasite segments were less than 3000 μm^2^ and the total numbers of parasites were divided into quartiles by area.

### Generation of *P. falciparum fabi*^−^ parasites

Targeting sequences 5′ and 3′ to *P. falciparum FabI* (PFF0730c) were cloned into plasmid pCC1 to facilitate positive–negative selection (Maier *et al*., 2006) (Fig. S4). Restriction sites in the multiple cloning site were SacII/SpeI for the 5′ flank and AvrII/EcoRI for the 3′ flank. Sequencing was performed to confirm inserts and primers used are detailed in Table S2. Plasmid DNA was extracted by maxi prep kit (Qiagen). The NF54 line *P. falciparum* parasites were synchronized at ring stage with sorbitol 2 days prior to transfection. On the following day trophozoites were selected for WT cytoadherence properties by incubation in RPMI plus Gelofusine (Braun). Transfection of *P. falciparum* ring stages with 100 μg of DNA was performed by electroporation at 0.31 kV and 950 μF with a Bio-Rad Gene Pulser (Bio-Rad, La Jolla, CA). Cultures were placed on the positive selection drug WR99210 (Jacobus Pharmaceuticals, Princeton, NJ) 6 h post transfection and maintained as described (Crabb *et al*., 2004). This was followed by negative selection against the cytosine deaminase/uracil phosphoribosyl transferase gene product with 5-fluorocytosine in order to obtain a parental line with double cross-over homologous recombination, which results in specific *FabI* gene deletion.

Two individual clones with a *FabI* deletion were isolated (E6 and G8) and genotypic analysis was confirmed by Southern Blot (Fig. S4). Genomic DNA from WT NF54 and knockout lines was digested for 2–16 h with the following enzymes: 5′ test: KpnI/BglII and 3′ test: BglII/BamHI. Digested DNA was run on a 1% TAE agarose gel at 15 V for 18 h and transferred to Hybond-N membrane (Amersham) overnight at room temperature, UV cross-linked and pre-hybridized with herring sperm DNA for 2.5 h. A digoxygenin-labelled probe was prepared by PCR per supplier protocol (Roche) using the cloning primers. Hybridization was carried out for 18 h at 55°C. The blot was exposed to film for 10–60 min and developed per standard protocol.

### Assessment of *P. falciparum* growth

Parasite cultures of both the WT NF54 and the *fabi*^−^ clone E6 containing mainly ring stages were synchronized twice within 4 h using sorbitol (Lambros and Vanderberg, 1979). Parasite density was determined and the culture was diluted to 0.8% parasitaemia, 5% haematocrit. Cultures were maintained under an atmosphere of reduced oxygen at 37°C and medium was refreshed every 24 h. Growth was monitored by Giemsa-stained thin blood smears every 24 h and for each determination of percentage parasitaemia, the number of infected erythrocytes per at least 2000 erythrocytes was recorded.

## Supporting Information

**Fig. S1.** Integration of a second copy of *FabI* fused to a quadruple myc tag into the *P. yoelii* genome (PyFabI-myc). A. The *FabI* gene, including 1 kb upstream of the start methionine and up to but not including the stop codon was amplified and ligated upstream of the quadruple myc tag (4× myc) in the 4× myc tag integration vector. Following linearization of the vector with BsaI, the construct was transfected into *P. yoelii* blood stage schizonts which were subsequently injected into mice. The vector contains the *T. gondii DHFR/TS* mutated gene as a pyrimethamine selectable marker (TgDHFR) and integrants were selected for by pyrimethamine treatment. B. Ethidium bromide-stained agarose gel showing the integration of the FabI-myc vector into the *P. yoelii* genome. Only PyFabI-myc is positive for this test (‘int test’), whereas, as expected, both PyFabI-myc and wild-type parasite genomic DNA is positive for the *FabI*-specific open reading frame test (‘ORF test’). Note that the selected parasites have two copies of *FabI*, the wild-type copy and the 4× myc tagged copy, both with their endogenous promoter. PyFabI-myc should express a second copy of FabI with a myc epitope and the expression of the tagged protein should mimic that of the endogenous copy. The same integration strategy was used to generate PyFabG-myc and PyFabZ-myc..

**Fig. S2.** Lack of expression of quadruple-myc epitope-tagged FabI in the (A) developing midgut oocyst sporozoites and (B) free midgut sporozoites of the transgenic *P. yoelii* parasite PyFabImyc. PyFabI-myc was generated to express a second copy of FabI under the control of its endogenous promoter with a C-terminal quadruple-myc tag. Expression of FabI in PyFabI-myc was monitored by immunofluorescence assay (IFA) using a rabbit anti-myc antibody. The oocyst and midgut sporozoites were detected with a mouse anti-circumsporozoite protein (CSP) antibody. Fluorescent staining was achieved with Alexa Fluorconjugated secondary antibodies (Alexa Fluor 488, green and Alexa Fluor 594, red) specific to rabbit and mouse IgG. Nuclear staining was achieved with 4′,6-diamidino-2-phenylindole (DAPI). Differential interference contrast (DIC) and fluorescent images were captured and processed using deconvolution microscopy and a merge of the captured images is presented on the far right pane (merge). Scale bar is 5 μm. FabI-myc expression was not detectable during sporozoite development in the mosquito.

**Fig. S3.** Successful deletion of *P. yoelii FabB/F* and *FabZ* by double cross-over homologous recombination to generate *P. yoelii fabb/f* ^−^ and *fabz* ^−^ knockout parasites. (A) DNA fragments of approximately 800 base pairs spanning the 5′ and 3′ UTR of the *FabB/F* gene (PY04452) and *FabZ* gene (PY01586) were ligated into the b3D.DT^H.^D vector. The vector contains the *T. gondii DHFR/TS* mutated gene as a pyrimethamine selectable marker (TgDHFR). The vector was linearized with KpnI and SacII and transfected into *P. yoelii* blood stage schizonts which were then injected into SW mice. Double cross-over homologous recombination was selected for with pyrimethamine and con- firmed by a positive PCR result using the primer sets ‘test 1’ and ‘test 2’ and a negative PCR result for the deleted gene (‘wt test’). (B) Ethidium bromide-stained agarose gel showing PCR products from the amplification of parasite genomic DNA from two clonal populations (clone 1 and clone 2) of the *FabB/F* gene deletion and wild-type (wt). (C) Similar to (D) but for the *FabZ* gene. The PCR results demonstrate the successful deletion of the genes because the ‘test 1’ and ‘test 2’ primer sets are positive only for the knockout population. Similarly, the wild-type test (‘wt test’) is positive only for the wild-type population. The result shows that the *FabB/F* and *FabZ* genes have been successfully deleted from blood stage *P. yoelii* parasites.

**Fig. S4.** Deletion of *FabI* in *P. falciparum*. (A) Targeting sequences 5′ and 3′ to *P. falciparum FabI* (PFF0730c) were cloned into plasmid pCC1 to facilitate positive-negative selection (Maier *et al.*, 2006). Restriction sites in the multiple cloning site were SacII/SpeI for the 5′ flank and AvrII/EcoRI for the 3′ flank. Genomic DNA from WT NF54 and knockout lines (clones E6 and G8) were assayed by Southern Blot and using the 5′ and 3′ flanks as probes and the expected restriction fragments resulting from enzymatic digestion of the WTloci and KO loci are shown. (B) The fragments recognized by the 5′ probe of KpnI/BglII restricted DNA are 6.1 kb for the WT locus and 1.3 kb for the KO locus. (C) The fragments recognized by the 3′ probe of BgllI/BamHI restricted DNA are 4.6 kb for the WT locus and 4.1 kb for the KO locus.

**Table S1.** *P. yoelii fabb/f* ^−^ and *fabz* ^−^ parasites develop normally in the mosquito.

**Table S2.** Oligonucleotide primers used in the study.
