# Supplementary material for: Type II fatty acid synthesis is essential only for malaria parasite late liver stage development
Source: Cell Microbiol. 2008 Dec 18;11(3):506–20. doi: 10.1111/j.1462-5822.2008.01270.x (PMC2688669; doi:10.1111/j.1462-5822.2008.01270.x)

Supplementary Information

Supplementary Table 1

***P. yoelii fabb/f*$^{-}$ and *fabz*$^{-}$ parasites develop normally in the mosquito**

	wildtype (n=4) mean +/- SD	*fabb/f*$^{-}$ clone 1 (n=4) mean +/- SD	*fabz*$^{-}$ clone 1 (n=4) mean +/- SD
# salivary gland sporozoites	10384 +/- 3803	8890 +/- 3565	6903 +/- 3167

Blood stage parasites of *fabb/f*$^{-}$ clone 1 and *fabz*$^{-}$ clone 1 were maintained in Swiss Webster mice and fed to *Anopheles stephensi* mosquitoes when parasitemia was approximately 1.5% and abundant exflagellation was detected. At day 10 post-infectious blood meal, parasite midgut infection was checked by dissecting 20 mosquitoes. In all cycles for both parasite strains, midgut infections were seen in over 80% of mosquitoes. On day 14 post-infection, salivary gland sporozoites were removed and the average number of sporozoites per mosquito was determined. There was no significant difference in the number of salivary gland sporozoites from wildtype, *fabb/f*$^{-}$ or *fabz*$^{-}$ parasites.

Supplementary Table 2

<u>Oligonucleotide primers used in the study.</u>

Primers used to generate PyFabI-myc.

Forward primer: TAccgcggTAGAACAAAAGACATACCACCG

Reverse primer: ATactagtTGAGTCCGAAGCTTGAAATAAAT

Primers used to generate PyFabZ-myc.

Forward primer: TAccgcggAATTGAGCAAAAGATATTATCTCC

Reverse primer: ATactagtTTTTGACATTGCAAAAATCATATTC

Primers used to generate PyFabG-myc.

Forward primer: TAccgcggATCATAAAGCGATAAAATTAACCC

Reverse primer: TAtctagaACTTGATAATCCACCATCAATTAT

Primers used to generate *P. yoelii fabb/f*$^{-}$.

Primers for UTRs

5'UTR forward primer: ATggtaccCATTTTAAACAATCAAATGATATAAG

5'UTR reverse primer: ATATaagcttAAGAATATTTTTAAGGGCCATTTC

3'UTR forward primer: ATgcggccgcAAGTGCATGTGCAACATCAGG

3'UTR reverse primer: ATATccgcggATCAACTAATAAATGATAATATCAAC

Primers to test for recombination

Test 1 forward primer: CGCGAACAAACCGCTCATGC

Test 1 reverse primer: GTGCTGCAAGGCGATTAAGT

Test 2 forward primer: TCAATGATTCATAAATAGTTGGACTTG

Test 2 reverse primer: GTGTGCGAGAAAGAATGTCATG

Primers to test for wildtype

Wildtype forward primer: TTATTGGAAGTGGTATTGGTGG

Wildtype reverse primer: ATCAACTAATAAATGATAATATCAAC

Primers used to generate *P. yoelii fabz*$^{-}$.

Primers for UTRs

5'UTR forward primer: ATggtaccGATATTATCTCCTTATTAATACGTG

5'UTR reverse primer: ATATaagcttGTGTATCACACATACTAATCAATG

3'UTR forward primer: ATATactagtAATGTCAAAATGATATAAATATAAGG

3'UTR reverse primer: ATccgcggTGTGCTTGTATTTTTATTGGACC

Primers to test for recombination

Test 1 forward primer: TTTATGTATCTGAAGTTACAACTG

Test 1 reverse primer: GTGCTGCAAGGCGATTAAGT

Test 2 forward primer: TCAATGATTCATAAATAGTTGGACTTG

Test 2 reverse primer: ATGAACATATGCACATATGTTCG

Primers to test for wildtype

Wildtype forward primer: TGAAAAGGATGATAATATTAGTGC

Wildtype reverse primer: GCCATTAAAAAAATGTTCATTTGC

Primers used to generate *P. falciparum fabi*$^{-}$.

Primers for UTRs

5'UTR forward primer: atcccgcggGAGTTTATTCATGTGGACATGTGC

5'UTR reverse primer:

atcactagtATAACTTCGTATAGCATACATTATACGAAGTTATgttcattctgaag aacgttatgg

3'UTR forward primer:

atcgaattcATAACTTCGTATAATGTATGCTATACGAAGTTATtgacgcttcttttg atactgc

3'UTR reverse primer: actcctaggATCCACATATATTGTCTGCC

Primers used for real-time PCR

Primer Sequence

18 S rRNA F	5’ GGGGATTGGTTTTGACGTTTTTGCG 3’
18 S rRNA R	5’ AAGCATTAAATAAAGCGAATACATCCTT 3’
PY01841F	5’AAGCATCGAATTTTGCTCAAGAA 3’
PY01841R	5’GGGTGAGTTGAAGGAAGTTCATTT 3’
PY06134F	5’GAAGATATAGATAAAACGCCGGAAGA 3’
PY06134R	5’TCCAGGGCAATCAATATGACTATAAT 3’
PY01586F	5’GTAGATGGTGTTAAATGGAAGAAACCT 3’
PY01586R	5’CATATTCTCGATTTCTATTACTACATGATTACC 3’
PY02416F	5’CGATCCATTGCGAAAACGTT 3’
PY02416R	5’CAGTTGCTTTATACCCAAGGGAAT 3’
PY03846F	5’GTAAATTTATGAATCCGGGAGGAA 3’
PY03846R	5’ATGCAGCTTTTGCACTTGACA3’
PY04452F	5’ TGTGGAGGAACTGAAGCTAGTGTT3’
PY04452R	5’AACAGCCATTGCTCTTAATGCA3’

Supplementary Figure Legends

Figure S1: Integration of a second copy of *FabI* fused to a quadruple myc tag into the *P. yoelii* genome (PyFabI-myc). a. The FabI gene, including one kilobase upstream of the start methionine and up to but not including the stop codon was amplified and ligated upstream of the quadruple myc tag (4x myc) in the 4x myc tag integration vector. Following linearization of the vector with *BsaI*, the construct was transfected into *P. yoelii* blood stage schizonts which were subsequently injected into mice. The vector contains the *T. gondii* DHFR/TS mutated gene as a pyrimethamine selectable marker (*TgDHFR*) and integrants were selected for by pyrimethamine treatment. b. Ethidium bromide stained agarose gel showing the integration of the FabI-myc vector into the *P. yoelii* genome. Only PyFabI-myc is positive for this test ('int test'), whereas, as expected, both PyFabI-myc and wildtype parasite genomic DNA is positive for the FabI specific open reading frame test ('ORF test'). Note that the selected parasites have two copies of FabI, the wildtype copy and the 4x myc tagged copy, both with their endogenous promoter. PyFabI-myc should express a second copy of FabI with a myc epitope and the expression of the tagged protein should mimic that of the endogenous copy. The same integration strategy was used to generate PyFabG-myc and PyFabZ-myc.

Figure S2: Lack of expression of quadruple-myc epitope-tagged FabI in the (a) developing midgut oocyst sporozoites and (b) free midgut sporozoites of the transgenic *P. yoelii* parasite PyFabI-myc. PyFabI-myc was generated to express

a second copy of FabI under the control of its endogenous promoter with a C-terminal quadruple-myc tag. Expression of FabI in PyFabI-myc was monitored by immunofluorescence assay (IFA) using a rabbit anti-myc antibody. The oocyst and midgut sporozoites were detected with a mouse anti-circumsporozoite protein (CSP) antibody. Fluorescent staining was achieved with Alexa Fluor-conjugated secondary antibodies (Alexa Fluor 488, green and Alexa Fluor 594, red) specific to rabbit and mouse IgG. Nuclear staining was achieved with 4', 6-diamidino-2-phenylindole (DAPI). Differential interference contrast (DIC) and fluorescent images were captured and processed using deconvolution microscopy and a merge of the captured images is presented on the far right pane (merge). Scale bar is 5 μm. FabI-myc expression was not detectable during sporozoite development in the mosquito.

Figure S3: Successful deletion of *P. yoelii FabB/F* and *FabZ* by double cross-over homologous recombination to generate *P. yoelii fabb/f* ⁻ and *fabz*⁻ knockout parasites. (a) DNA fragments of approximately 800 base pairs spanning the 5' and 3' UTR of the *FabB/F* gene (PY04452) and *FabZ* gene (PY01586) were ligated into the b3D.DT^H.^D vector. The vector contains the *T. gondii DHFR/TS* mutated gene as a pyrimethamine selectable marker (*TgDHFR*). The vector was linearized with *KpnI* and *SacII* and transfected into *P. yoelii* blood stage schizonts which were then injected into SW mice. Double crossover homologous recombination was selected for with pyrimethamine and confirmed by a positive PCR result using the primer sets 'test 1' and 'test 2' and a negative PCR result

for the deleted gene (‘wt test’). (b) Ethidium bromide stained agarose gel showing PCR products from the amplification of parasite genomic DNA from two clonal populations (clone 1 and clone 2) of the *FabB/F* gene deletion and wildtype (wt). (c) Similar to (b) but for the *FabZ* gene. The PCR results demonstrate the successful deletion of the genes since the ‘test 1’ and ‘test 2’ primer sets are positive only for the knock-out population. Similarly, the wildtype test (‘wt test’) is positive only for the wildtype population. The result shows that the FabB/F and FabZ genes have been successfully deleted from blood stage *P. yoelii* parasites.

Figure S4: Deletion of *FabI* in *P. falciparum*. (a) Targeting sequences 5’ and 3’ to *P. falciparum* FabI (PFF0730c) were cloned into plasmid pCC1 to facilitate positive-negative selection (Maier *et al.*, 2006). Restriction sites in the multiple cloning site were SacII/SpeI for the 5’ flank and AvrII/EcoRI for the 3’ flank. Genomic DNA from WT NF54 and knockout lines (clones E6 and G8) were assayed by Southern Blot and using the 5’ and 3’ flanks as probes and the expected restriction fragments resulting from enzymatic digestion of the WT loci and KO loci are shown. (b) The fragments recognized by the 5’ probe of KpnI/BglII restricted DNA are 6.1 kb for the WT locus and 1.3 kb for the KO locus. (c) The fragments recognized by the 3’ probe of BglII/BamHI restricted DNA are 4.6 kb for the WT locus and 4.1 kb for the KO locus.

Supplementary Figure 1

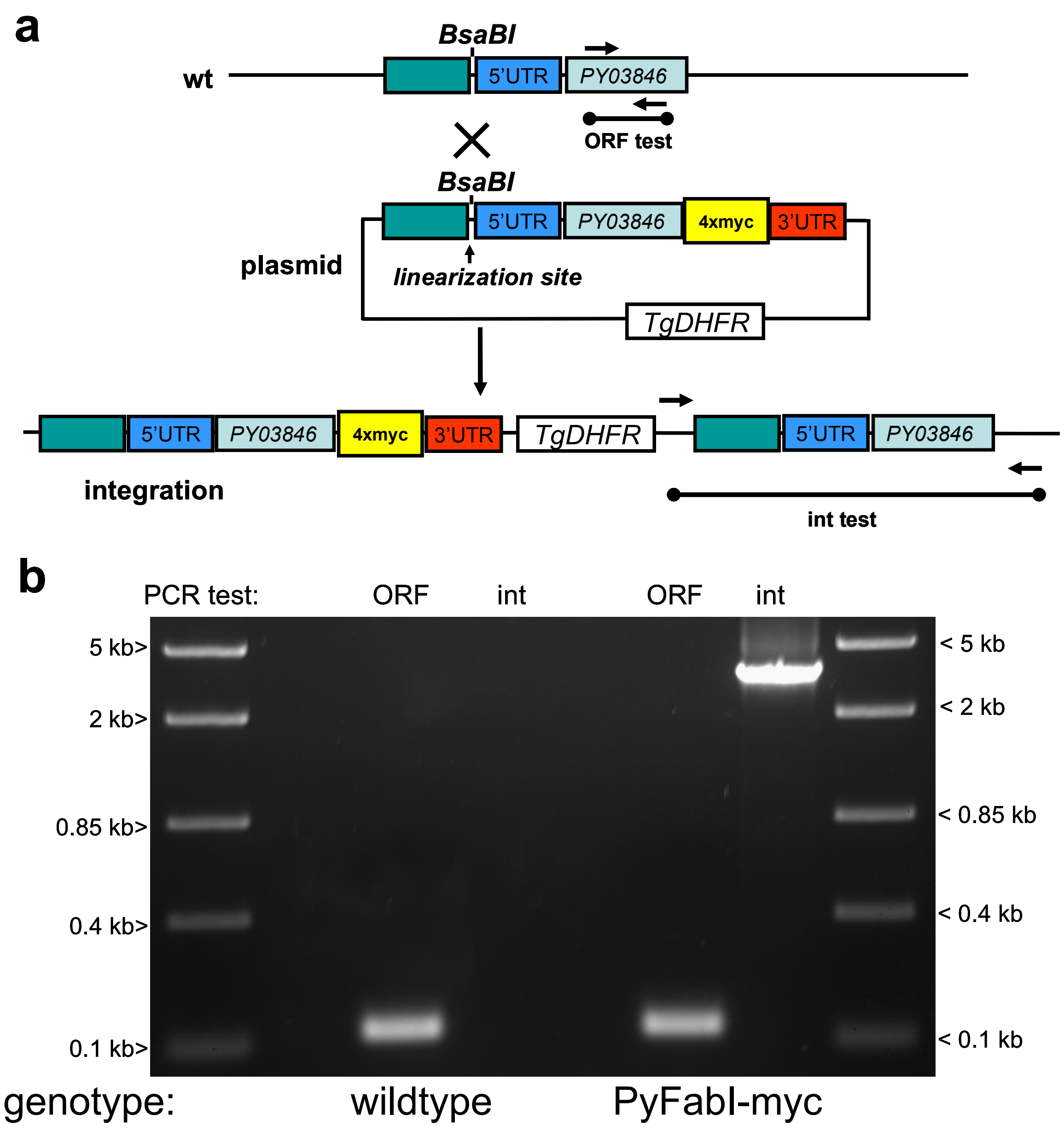

Supplementary Figure 2

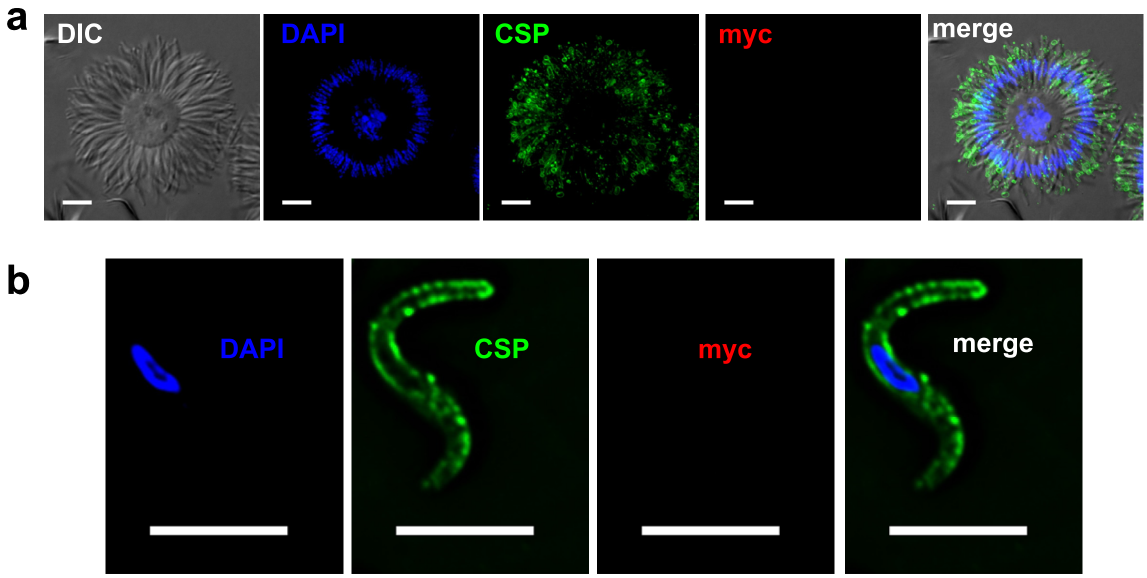

Supplementary Figure 3

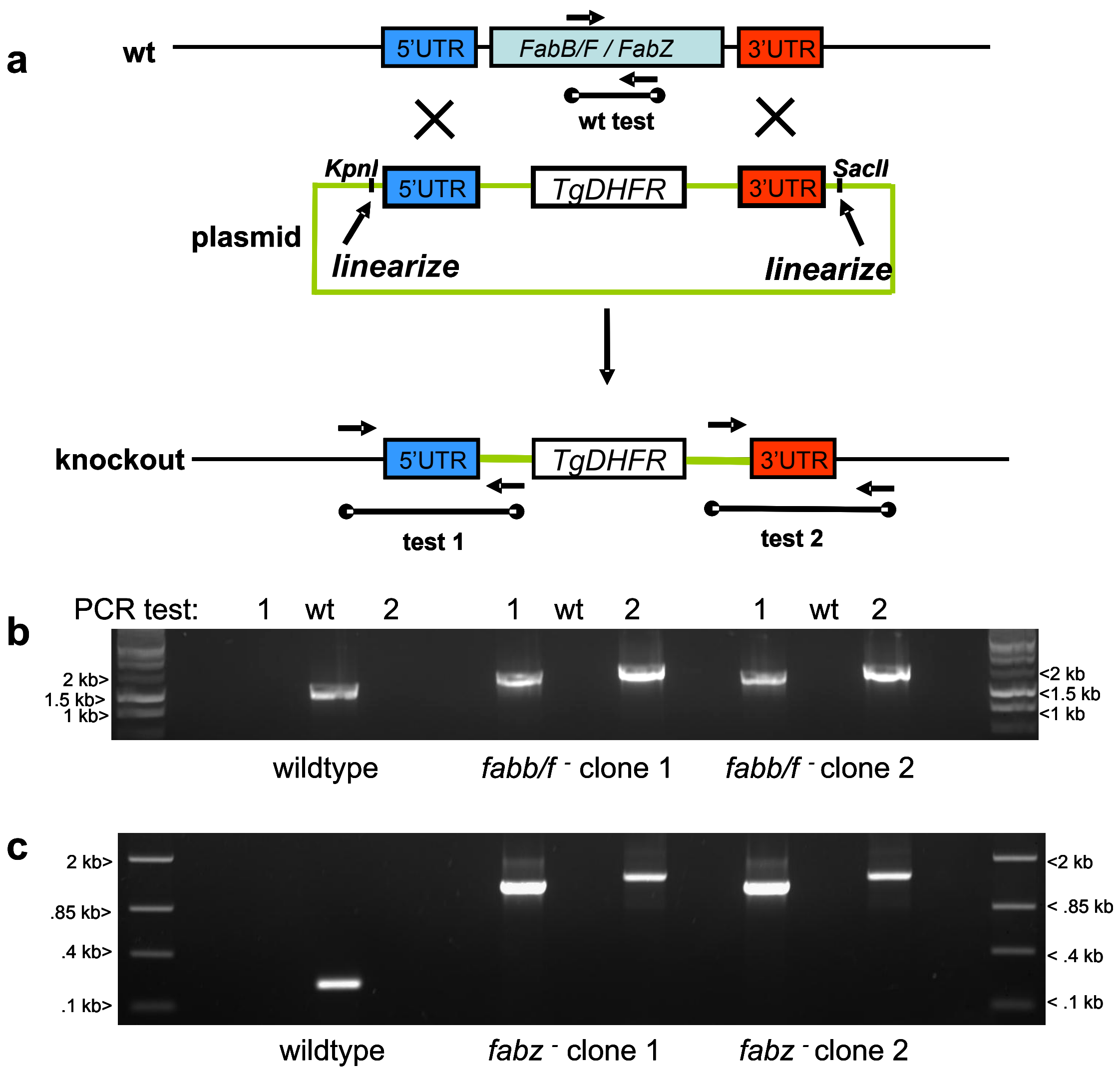

Supplementary Figure 4

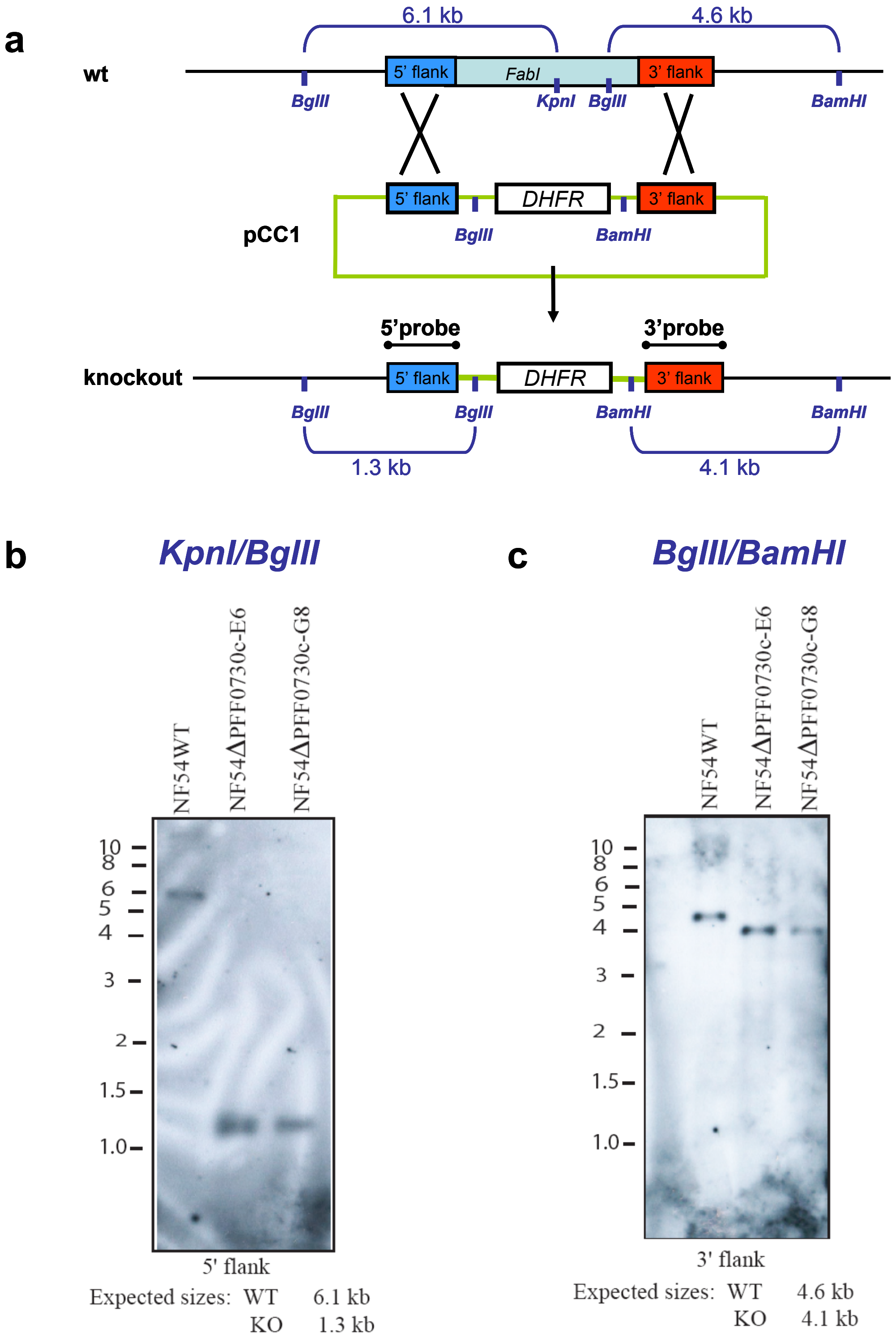

Supplement: Supplementary file 1 [file cmi0011-0506-SD1.pdf]
